# Supplementary material for: Deep learning-assisted co-registration of full-spectral autofluorescence lifetime microscopic images with H&E-stained histology images
Source: Commun Biol. 2022 Oct 21;5:1119. doi: 10.1038/s42003-022-04090-5 (PMC9586936; doi:10.1038/s42003-022-04090-5)
Supplement: Supplementary file 2 — Supplementary Information [file 42003_2022_4090_MOESM2_ESM.pdf]

Deep Learning-Assisted Co-registration of Full-Spectral Fluorescence Lifetime Microscopic Images with H&E-Stained Histology Images

Qiang Wang[1,*], Susan Fernandes[1], Gareth O. S. Williams[1], Neil Finlayson[2], Ahsan R. Akram[1], Kevin Dhaliwal[1], James R. Hopgood[3], and Marta Vallejo[4]

[1]Centre for Inflammation Research, Queen's Medical Research Institute, University of Edinburgh, Edinburgh, UK

[2]Institute for Integrated Micro and Nano Systems, School of Engineering, University of Edinburgh, Edinburgh, UK

[3]Institute for Digital Communications, School of Engineering, University of Edinburgh, Edinburgh, UK

[4]School of Engineering and Physical Sciences, Heriot-Watt University, Edinburgh, UK

[*]Corresponding: Q.Wang@ed.ac.uk

Supplementary Information

List of Figures

False-color FS-FLIM images from 500nm to 780nm. 3
False-color FS-FLIM images from 500nm to 780nm (cont.). 4
Extreme examples of the registration with lifetime images. 5
Ineffectiveness of multi-modality intensity-based registration approaches. 6
Stitching results at various emission wavelengths 7
Stitching results at various emission wavelengths (cont.) 8
Stitching results at various emission wavelengths (cont.) 9
Processing of histology images before being input into the CycleGAN. 10
Quantitative comparison of co-registration results by partial photometric loss. . 12
GUI of the software developed for the co-registration 13

List of Tables

Quantitative evaluation of the co-registration using intensity, lifetime, and false histology images 11

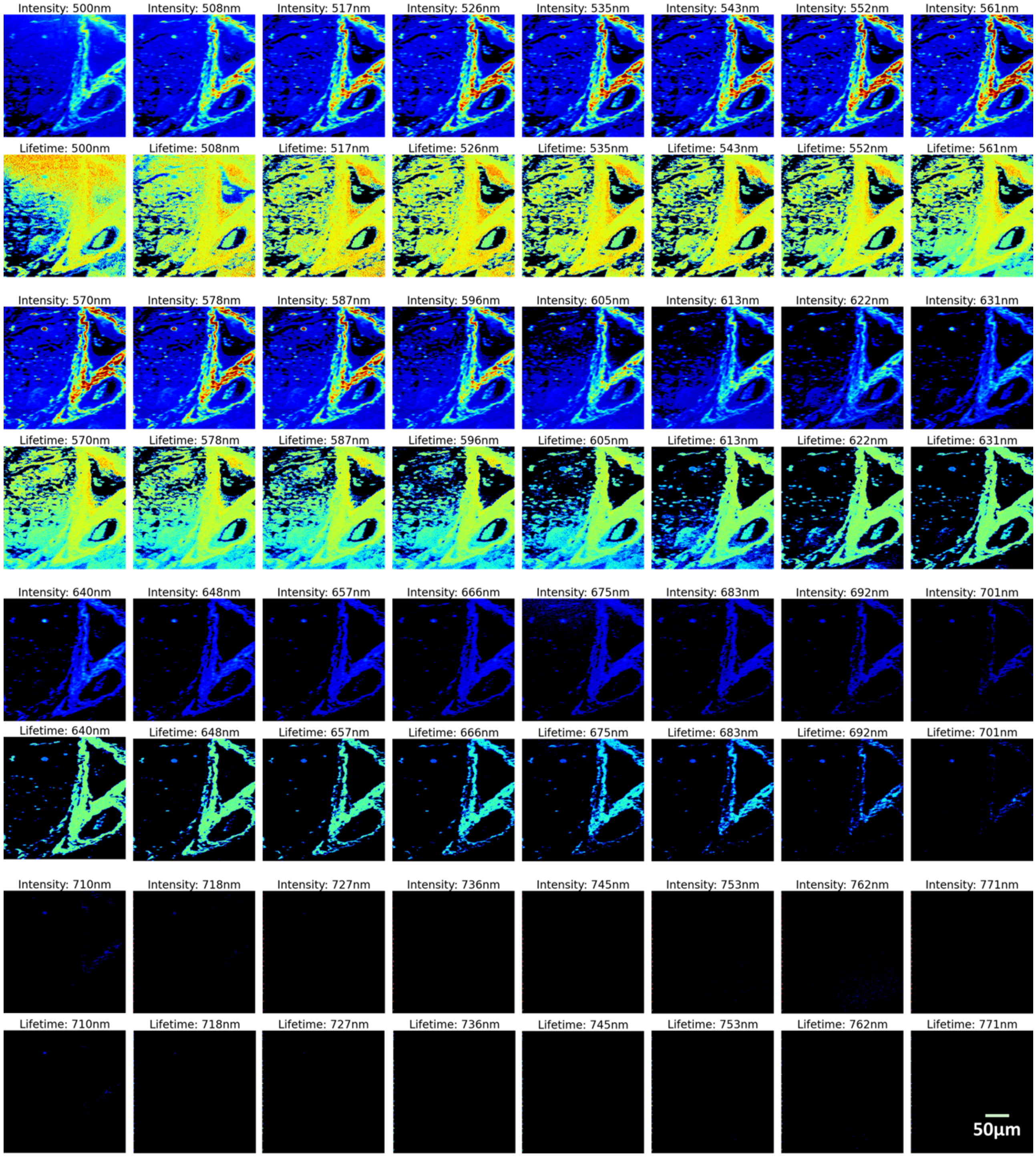

Supplementary Figure 1: False-color FS-FLIM images from 500nm to 780nm. False-color FS-FLIM images of 256x256 pixels, with a FOV of 600x600 μm, at the wavelengths from 500nm to 780nm. The scale of intensity images are fixed at [0, 1500], and the lifetime is at [$0.5\mu s$, $3\mu s$].

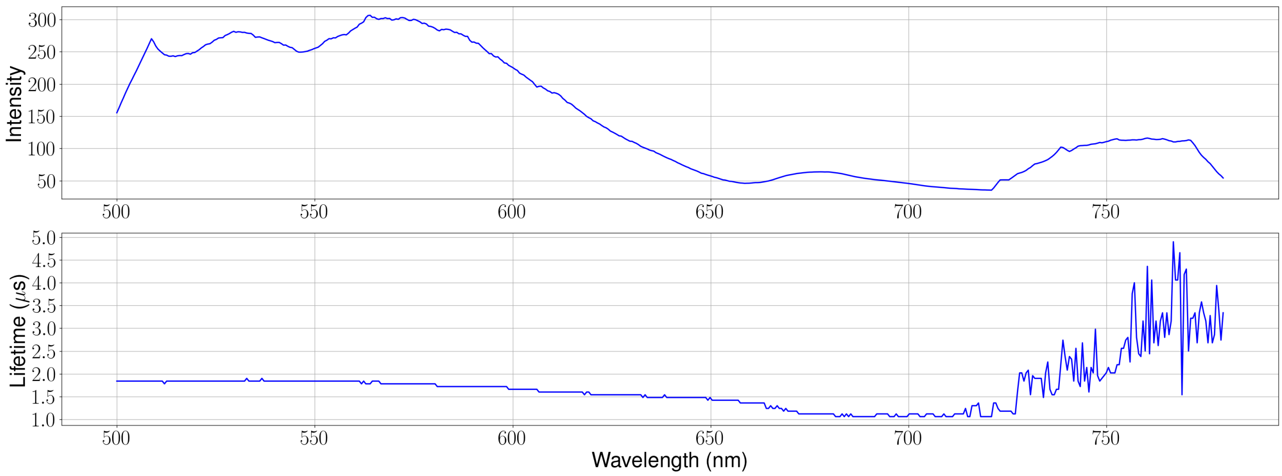

Supplementary Figure 1: False-color FS-FLIM images from 500nm to 780nm (cont.) Average intensity and histogram-averaged lifetime along wavelengths from 500nm to 780nm. When wavelength is in [500nm, 600nm], signal-to-noise ratio (SNR) is high enough for reliable reconstruction of lifetime images, and the images contain sufficient structural information. At the range of [600nm, 710nm], lifetime at some pixels is unable to be reconstructed as the SNR is below the limit. When wavelength is beyond 710nm, SNR dramatically deceases and hence the majority of pixel lifetime values are unable to be calculated reliably.

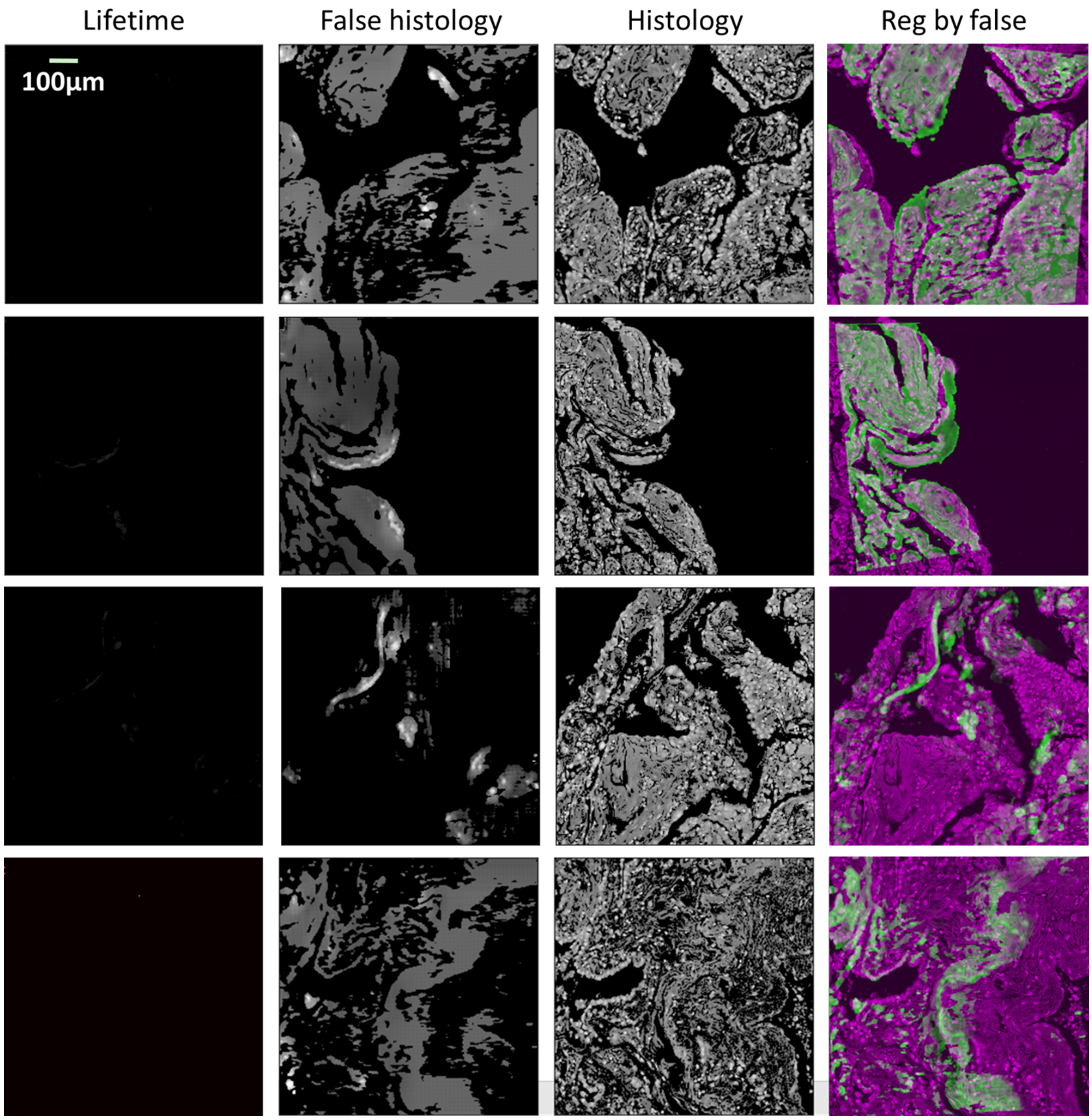

Supplementary Figure 2: Extreme examples of the registration with lifetime images. All lifetime images are with wavelengths over 710nm. In lifetime images (first column), there is little information on most pixels as SNR is below the threshold described in Equation (1) and (2). However, the translated images (second column) present some structural content. In some cases, the structure is enough for the regression (first and second rows), but others are not (third and fourth rows).

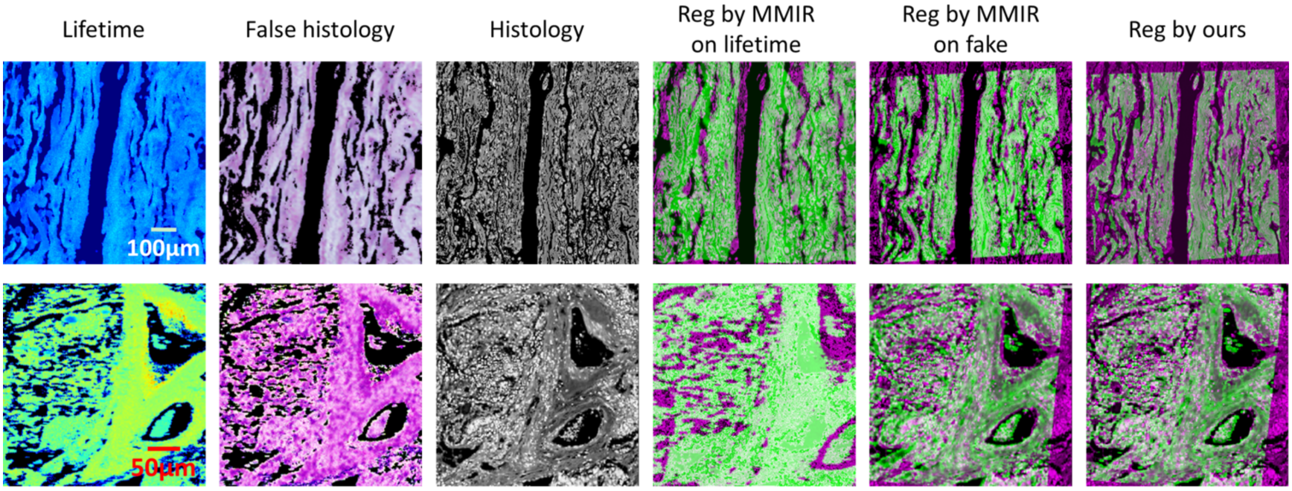

Supplementary Figure 3: Ineffectiveness of multi-modality intensity-based registration approaches performed directly on lifetime and the corresponding false histology images. A multi-scale intensity-based registration approach based on similarity is applied to both lifetime (first column) and false histology (second column) images and the results are on fourth and fifth column, respectively, in comparison with our approach (sixth column).

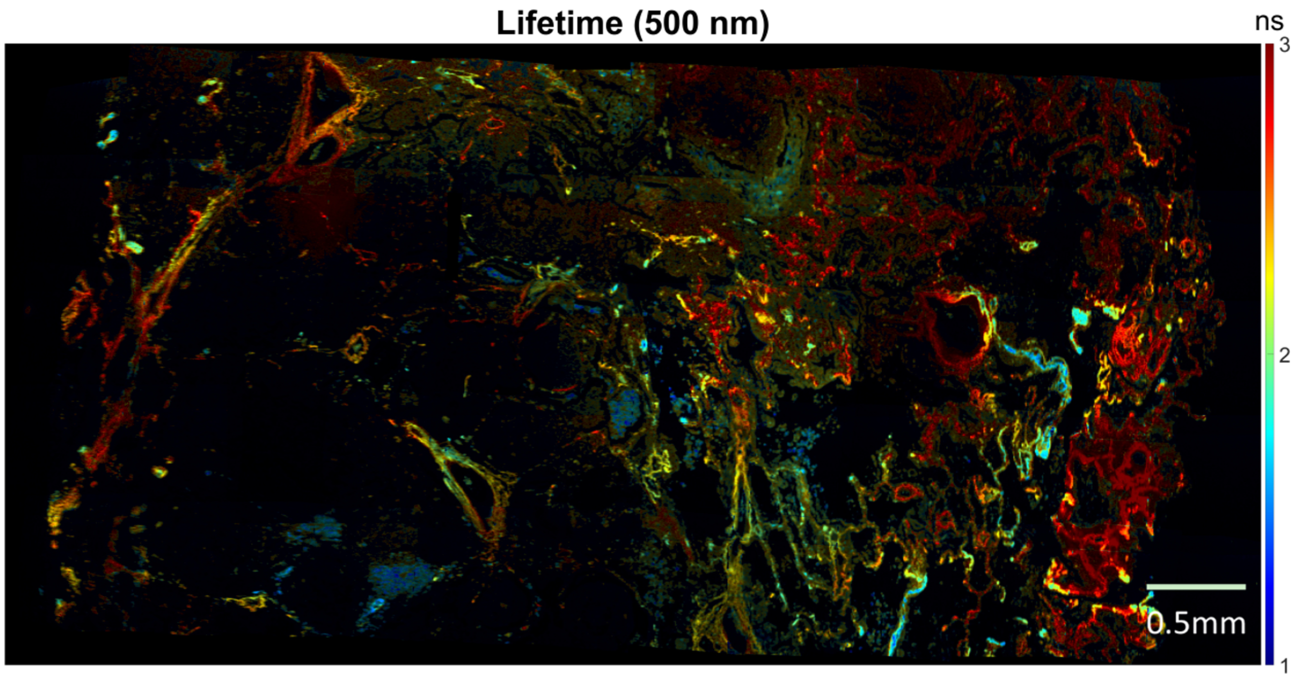

(a)

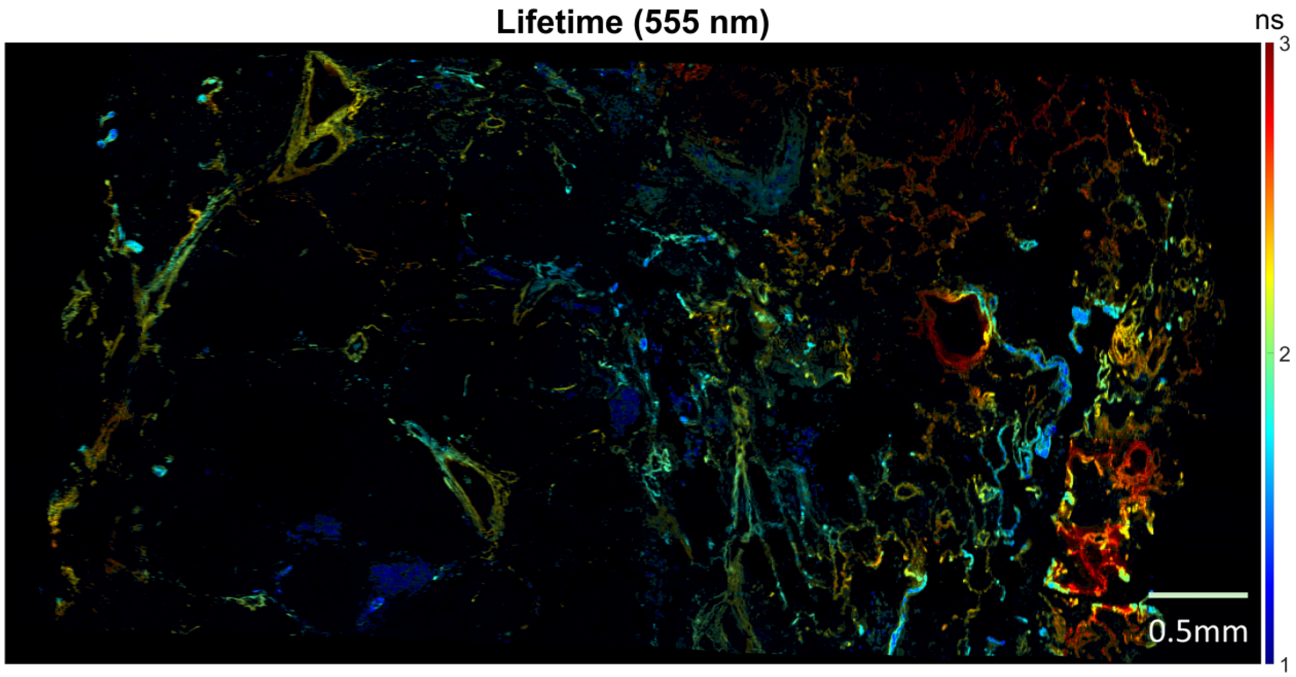

(b)

Supplementary Figure 4: Stitching results at various emission wavelengths. (a) 500nm, and (b) 555nm.

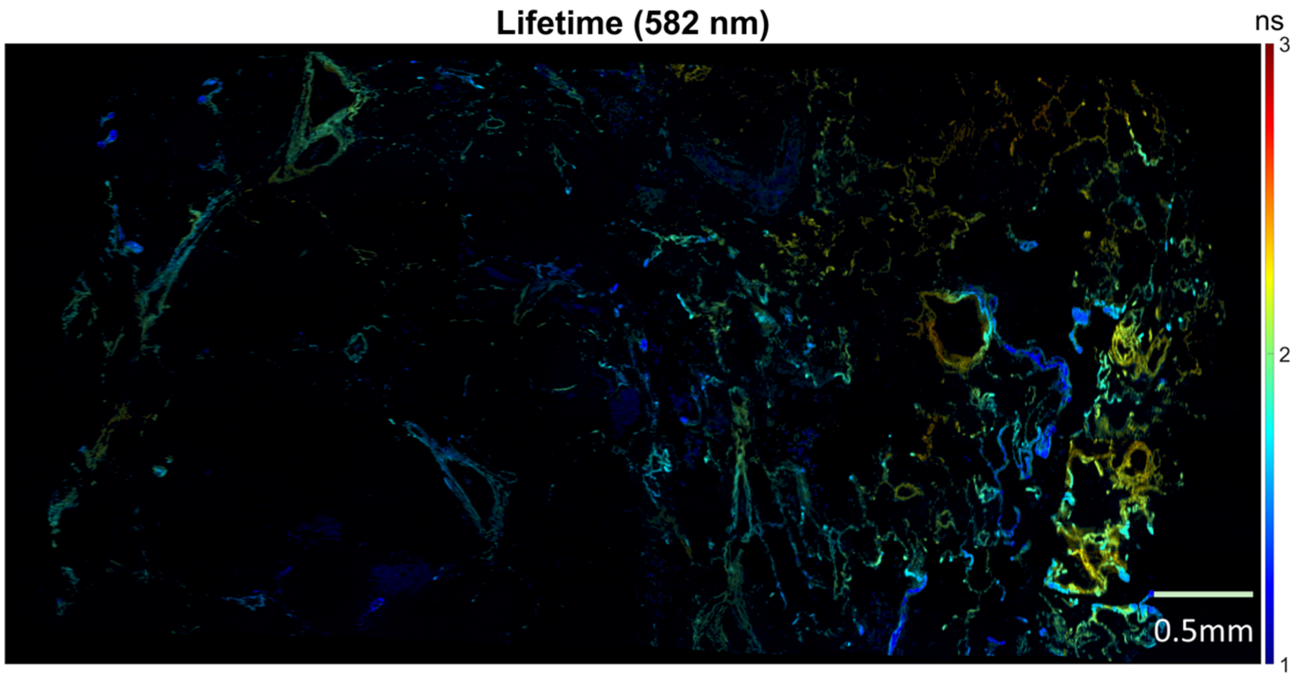

(c)

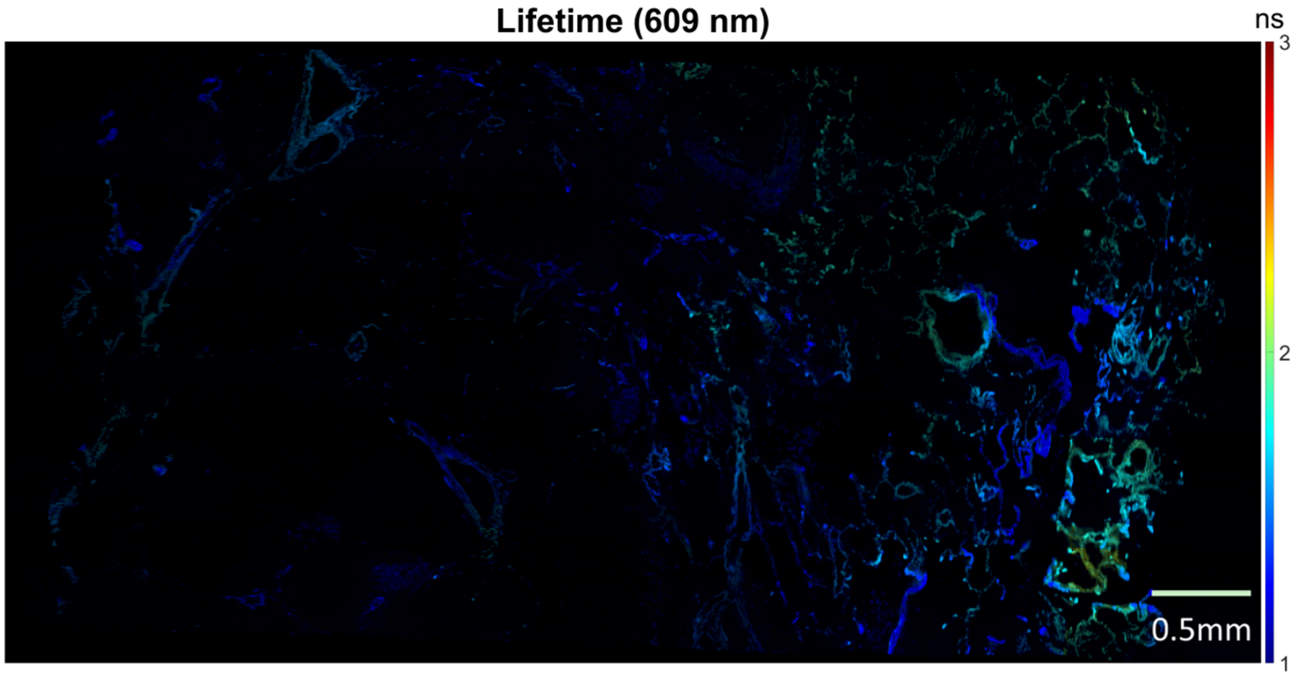

(d)

Supplementary Figure 4: Stitching results at various emission wavelengths (cont.). (c) 609nm, and (d) 637nm.

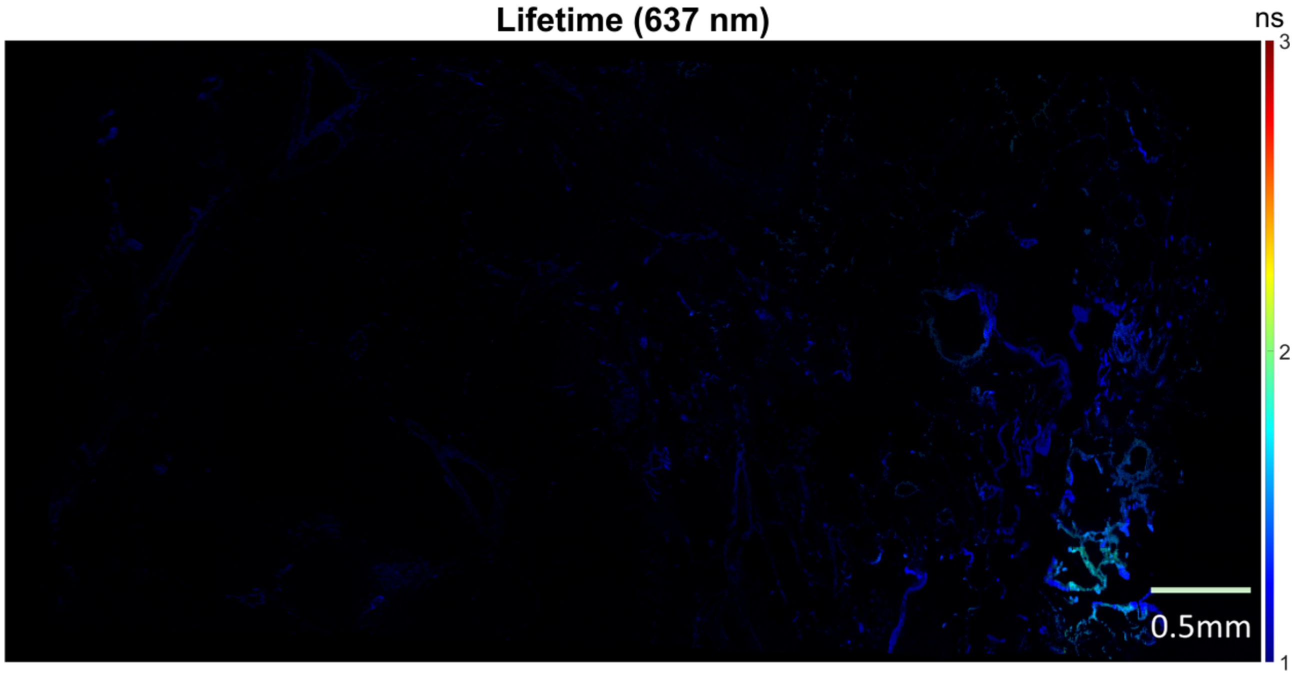

(e)

Supplementary Figure 4: Stitching results at various emission wavelengths (cont.). (e) 637nm.

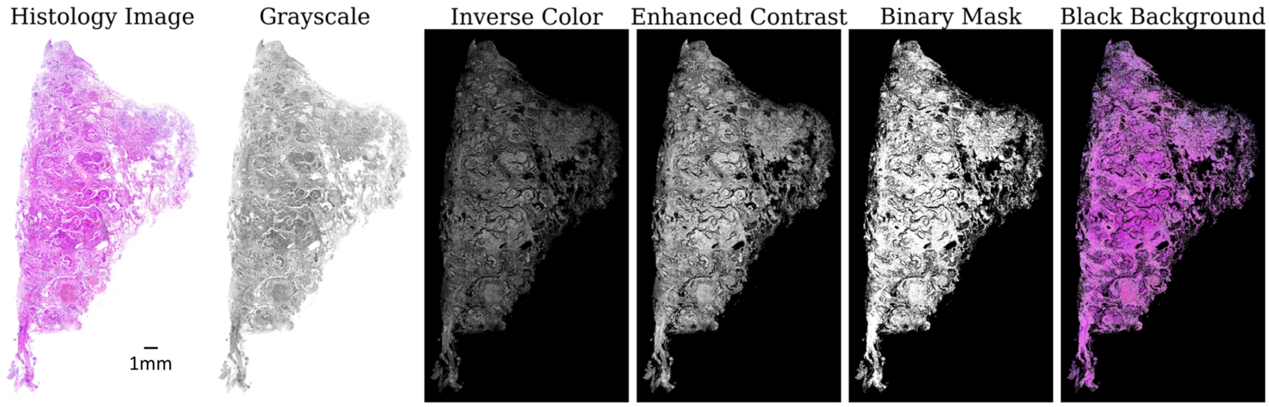

Supplementary Figure 5: Processing of histology images before being input into the CycleGAN.

	Similarity Metrics		
	**MSE**	**NMI**	**NCC**
**Intensity**	0.211±0.023	1.007±0.002	0.799±0.045
**Lifetime**	0.135±0.037	1.020±0.018	**0.857±0.106**
**false histology**	**0.122±0.035**	**1.024±0.021**	0.832±0.078

Table 1: Quantitative evaluation of the co-registration using intensity, lifetime, and false histology images. The best results are in **bold**. Values are presented in mean±standard deviation on mean squared error (MSE), normalised mutual information (NMI), and normalised cross-correlation (NCC). Smaller values for MSE are better, whereas larger values for NMI and NCC are better.

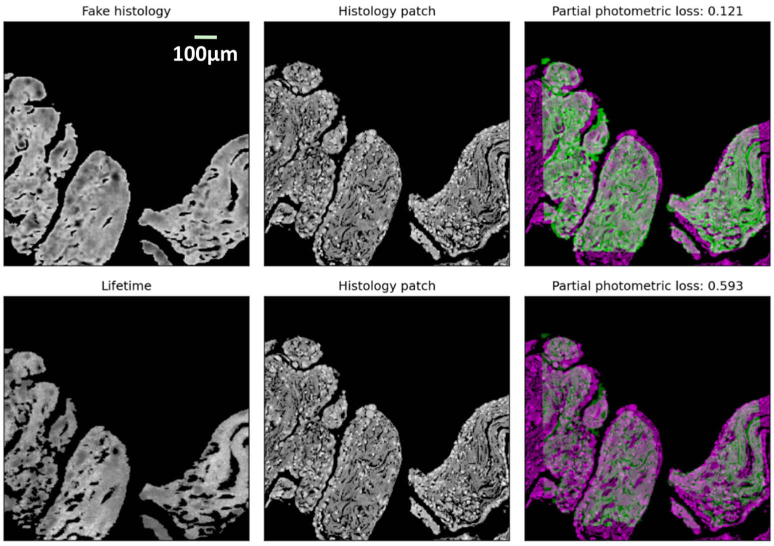

(a)

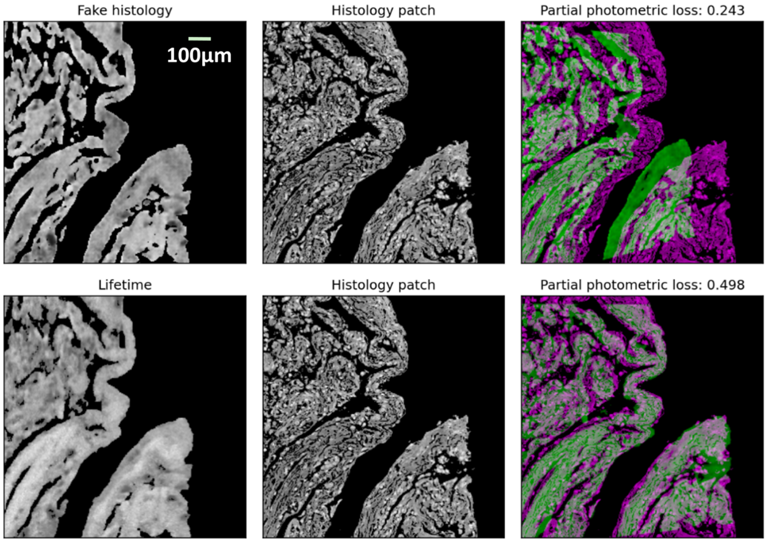

(b)

Supplementary Figure 6: Quantitative comparison of co-registration results by partial photometric loss. (a) both synthetic histology and lifetime can achieve reasonable co-registration, and (b) lifetime image presents a good registration result, whereas synthetic histology images does not. However, in both cases, the partial photometric losses per the fake histology images are lower than these by the lifetime images.

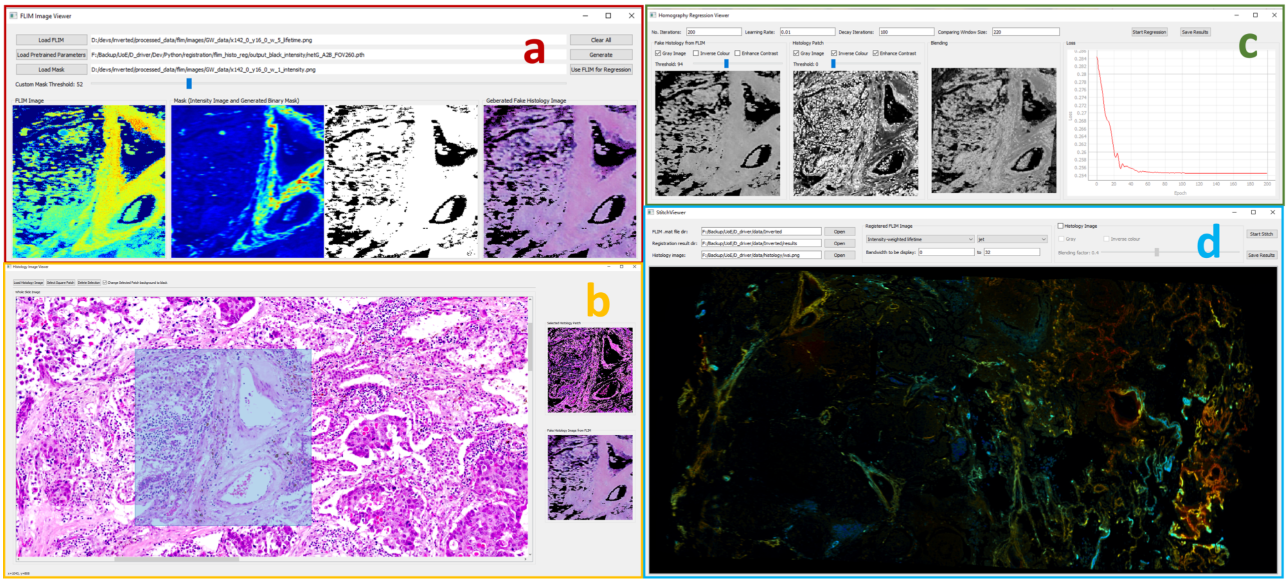

Supplementary Figure 7: GUI of the software developed for the co-registration, which is composed of four parts. (a) generates false histology images, (b) is used for the interactive crop of histology patches, (c) performs the proposed regression, and (d) produces the stitching of the registered results.
