# Supplementary material for: Deep learning-assisted co-registration of full-spectral autofluorescence lifetime microscopic images with H&E-stained histology images
Source: Commun Biol. 2022 Oct 21;5:1119. doi: 10.1038/s42003-022-04090-5 (PMC9586936; doi:10.1038/s42003-022-04090-5)
Supplement: Supplementary file 3 — Description of Additional Supplementary Files [file 42003_2022_4090_MOESM3_ESM.docx]

**Description of Additional Supplementary Files**

**File name:** Supplementary Data
**Description:** the source data for Figure 7.b
