# Supplementary material for: Deep learning-assisted co-registration of full-spectral autofluorescence lifetime microscopic images with H&E-stained histology images
Source: Commun Biol. 2022 Oct 21;5:1119. doi: 10.1038/s42003-022-04090-5 (PMC9586936; doi:10.1038/s42003-022-04090-5)
Supplement: Supplementary file 5 — Reporting Summary [file 42003_2022_4090_MOESM5_ESM.pdf]

nature portfolio

Corresponding author(s): Qiang Wang

Last updated by author(s): Sep 29, 2022

Reporting Summary

Nature Portfolio wishes to improve the reproducibility of the work that we publish. This form provides structure for consistency and transparency in reporting. For further information on Nature Portfolio policies, see our Editorial Policies and the Editorial Policy Checklist.

Statistics

For all statistical analyses, confirm that the following items are present in the figure legend, table legend, main text, or Methods section.

n/a	Confirmed	
☐	☒	The exact sample size (*n*) for each experimental group/condition, given as a discrete number and unit of measurement
☐	☒	A statement on whether measurements were taken from distinct samples or whether the same sample was measured repeatedly
☒	☐	The statistical test(s) used AND whether they are one- or two-sided<br>*Only common tests should be described solely by name; describe more complex techniques in the Methods section.*
☒	☐	A description of all covariates tested
☐	☒	A description of any assumptions or corrections, such as tests of normality and adjustment for multiple comparisons
☐	☐	A full description of the statistical parameters including central tendency (e.g. means) or other basic estimates (e.g. regression coefficient) AND variation (e.g. standard deviation) or associated estimates of uncertainty (e.g. confidence intervals)
☒	☐	For null hypothesis testing, the test statistic (e.g. *F*, *t*, *r*) with confidence intervals, effect sizes, degrees of freedom and *P* value noted<br>*Give P values as exact values whenever suitable.*
☒	☐	For Bayesian analysis, information on the choice of priors and Markov chain Monte Carlo settings
☒	☐	For hierarchical and complex designs, identification of the appropriate level for tests and full reporting of outcomes
☒	☐	Estimates of effect sizes (e.g. Cohen's *d*, Pearson's *r*), indicating how they were calculated

Our web collection on statistics for biologists contains articles on many of the points above.

Software and code

Policy information about availability of computer code

Data collection	Data was collected using MatLab (2019b) scripts that communicated with the detector FPGA via firmware interfacing throught the Opal Kelly frontpanel driver (version 4.5.6.0). The firmware bit-file enabled control of the on-PCB digital to analog converters which provides drive signals to the galvanometric mirrors. data was streamed from the image sensor to the FPGA to the PC running Mablab code. Retrieved data was entered into a four dimensional array (X, Y, Time bin, Spectral channel) which is saved as a Matlab file for lifetime reconstruction and image post-processing alongwith appropriate system perimeters such as selected image size and the length of the time bins determined by the "histmode" parameter with code provided through the University of Edinburgh DataShare services, https://doi.org/10.7488/ds/3099
Data analysis	The lifetime reconstruction code was analysed using Matlab (2019b) that take the 4D array created by the collection script as an input along with saved metadata, which is available through the University of Edinburgh DataShare service (https://doi.org/10.7488/ds/3099). The source code of CycleGAN for the translation from FS-FLIM images to synthetic histology images is available on https://junyanz.github.io/CycleGAN/. The software performed the registration mentioned in the "The Software" section is available on https://github.com/qiangwang57/coreg_flim_histology

For manuscripts utilizing custom algorithms or software that are central to the research but not yet described in published literature, software must be made available to editors and reviewers. We strongly encourage code deposition in a community repository (e.g. GitHub). See the Nature Portfolio guidelines for submitting code & software for further information.

Data

Policy information about availability of data

All manuscripts must include a data availability statement. This statement should provide the following information, where applicable:

- Accession codes, unique identifiers, or web links for publicly available datasets
- A description of any restrictions on data availability
- For clinical datasets or third party data, please ensure that the statement adheres to our policy

Data associated with figure 1, 2 (last 3 rows), 4, 6, 7, and 8 is available on the University of Edinburgh DataShare service (https://doi.org/10.7488/ds/3099). Data associated with figure 2 (first 2 rows), 3 and 5 is available on the University of Edinburgh DataShare service separately (https://doi.org/10.7488/ds/3421).

Human research participants

Policy information about studies involving human research participants and Sex and Gender in Research.

Reporting on sex and gender	This study does not distinguish sex and gender
Population characteristics	All patients involved in this study had a confirmed or suspected lung cancer of >3cm. However, this study concerns the co-registration, rather than the biological mechanisms, and thus, the population information was ignored as it would have little impact on the results.
Recruitment	Participants were recruited to the study if they had a confirmed or suspected lung cancer of >3cm. The training data was from pairs of normal and cancerous tissue from several patients, and the validation data presented in the paper represents early stage lung cancer to demonstrate the capability of label-free autofluorescence lifetime for differentiating cell types and variants. Although bias may exist during the training of CycleGAN, it would have little impact on the co-registration results.
Ethics oversight	A favourable ethical opinion was received from the South East of Scotland Research Ethics Service REC 1 (on behalf of the National Health Service), held by the NHS Lothian NRS BioResource (REC ref: 15/ES/0094).

Note that full information on the approval of the study protocol must also be provided in the manuscript.

Field-specific reporting

Please select the one below that is the best fit for your research. If you are not sure, read the appropriate sections before making your selection.

☒ Life sciences ☐ Behavioural & social sciences ☐ Ecological, evolutionary & environmental sciences

For a reference copy of the document with all sections, see nature.com/documents/nr-reporting-summary-flat.pdf

Life sciences study design

All studies must disclose on these points even when the disclosure is negative.

Sample size	The FS-FLIM data was acquired on 20 unstained lung samples, and the histology images used was collected from 40 stained lung samples.
Data exclusions	No data was excluded
Replication	The results were verified using the co-registration software for several times, which showed consistency.
Randomization	Data for training the CycleGAN was randomized for optimal training purpose, whereas data for validating purpose was not randomized as a common practice in deep learning.
Blinding	The investigators were blind to the samples used. However, the aim of this study is not to understand the biological mechanisms, but to co-register FS-FLIM image and the corresponding histology images, in assistance with deep learning technologies.

Reporting for specific materials, systems and methods

We require information from authors about some types of materials, experimental systems and methods used in many studies. Here, indicate whether each material, system or method listed is relevant to your study. If you are not sure if a list item applies to your research, read the appropriate section before selecting a response.

Materials & experimental systems

n/a	Involved in the study
☒	☐ Antibodies
☒	☐ Eukaryotic cell lines
☒	☐ Palaeontology and archaeology
☒	☐ Animals and other organisms
☒	☐ Clinical data
☒	☐ Dual use research of concern

Methods

n/a	Involved in the study
☒	☐ ChIP-seq
☒	☐ Flow cytometry
☒	☐ MRI-based neuroimaging
